# Feature- and Face-Exchange illusions: new insights and applications for the study of the binding problem

**DOI:** 10.3389/fnhum.2014.00804

**Published:** 2014-10-16

**Authors:** Arthur G. Shapiro, Gideon P. Caplovitz, Erica L. Dixon

**Affiliations:** ^1^Department of Psychology and Center for Behavioral Neuroscience, American UniversityWashington, DC, USA; ^2^Department of Psychology, University of Nevada, RenoReno, NV, USA

**Keywords:** motion perception, object perception, binding problem, visual periphery, animation, bouncing streaming illusions, illusion of causality

## Abstract

The binding problem is a longstanding issue in vision science: i.e., how are humans able to maintain a relatively stable representation of objects and features even though the visual system processes many aspects of the world separately and in parallel? We previously investigated this issue with a variant of the bounce-pass paradigm, which consists of two rectangular bars moving in opposite directions; if the bars are identical and never overlap, the motion could equally be interpreted as bouncing or passing. Although bars of different colors should be seen as passing each other (since the colors provide more information about the bars' paths), we found “Feature Exchange”: observers reported the paradoxical perception that the bars appear to bounce off of each other and exchange colors. Here we extend our previous findings with three demonstrations. “Peripheral Feature-Exchange” consists of two colored bars that physically bounce (they continually meet in the middle of the monitor and return to the sides). When viewed in the periphery, the bars appear to stream past each other even though this percept relies on the exchange of features and contradicts the information provided by the color of the bars. In “Face-Exchange” two different faces physically pass each other. When fixating centrally, observers typically report the perception of bouncing faces that swap features, indicating that the Feature Exchange effect can occur even with complex objects. In “Face-Go-Round,” one face repeatedly moves from left to right on the top of the monitor, and the other from right to left at the bottom of the monitor. Observers typically perceive the faces moving in a circle—a percept that contradicts information provided by the identity of the faces. We suggest that Feature Exchange and the paradigms used to elicit it can be useful for the investigation of the binding problem as well as other contemporary issues of interest to vision science.

## Introduction

The “binding problem” refers to the observation that the brain processes many aspects of the visual world separately and in parallel, yet we perceive a unified world, populated by coherent objects (James, 1890; Treisman, 1996; Holcombe et al, 2009). The implication is that the visual system binds together the output of separate processes (which presumably compute features, textures, colors, motion gradients, etc.) prior to creating our object-centric perceptual world. Two fundamental questions of the binding problem can be summarized as follows: (1) How, and under what conditions, does the brain combine (or fail to combine) the outputs of these separate processes to construct an object representation? (2) How are object representations maintained over time and space?

We recently examined the spatiotemporal conditions and the role feature-level processes play in representing and maintaining objects (Caplovitz et al., 2011) using a variant of the “bounce-pass paradigm” (Metzger, 1934; Michotte, 1946/1963; Kanizsa, 1969). In a typical version of the bounce pass paradigm, the interpretation of motion direction and object correspondence direction is intrinsically ambiguous, and the degree to which observers report one or the other of the potential percepts has been used to study a range of perceptual and cognitive processes. For example, versions of this basic paradigm have been used to study properties of cross-modal interactions and motion perception as well as object representations (Bertenthal et al., 1993; Watanabe and Shimojo, 1998; Sekuler and Sekuler, 1999; Mitroff et al., 2005; Feldman and Tremoulet, 2006).

**Figure 1:**
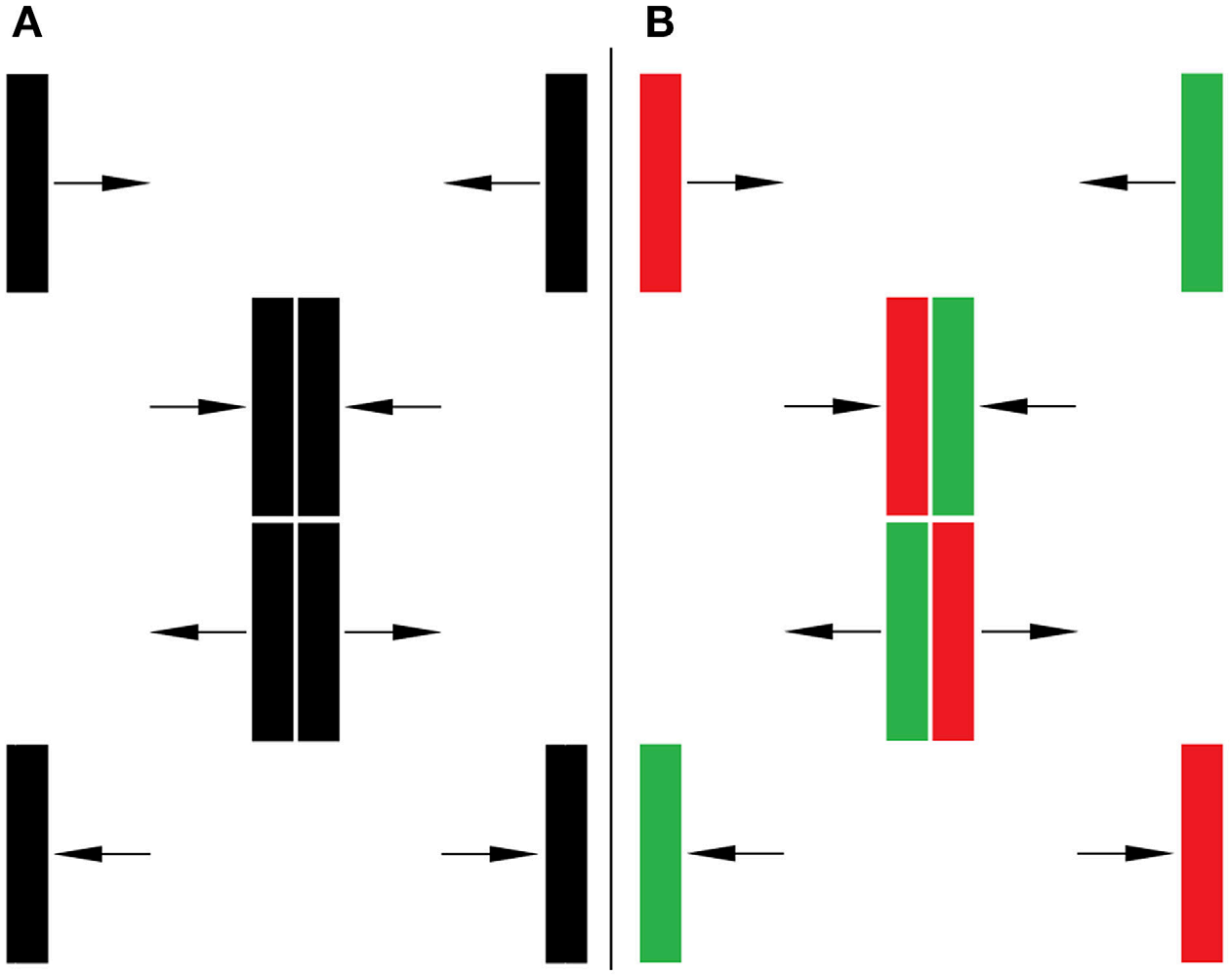
**Bounce/Stream Paradigm. (A)** Without and **(B)** with distinct features. Observers tend to perceive bouncing in both of these conditions, despite the fact that the colors in **(B)** are consistent with streaming.

The basic paradigm (illustrated in Figure 1A) consists of two rectangles; one that moves from right to left while the other moves from left to right. The display is ambiguous because the stimulus is wholly consistent with each rectangle passing from one side of the screen to the other (i.e., the perception of streaming) or as bouncing off of the other rectangle and returning to its point of origin (i.e., the perception of bouncing). If, at the point of intersection, one rectangle overlaps with the other rectangle observers will commonly perceive streaming (Sekuler and Sekuler, 1999). In our experiments, this potential cue is removed: at the critical point of intersection, the rectangles exactly exchange places and thus never have an overlapping edge. When the two rectangles are identical, the visual system usually resolves the ambiguity by producing the perception of bouncing (See Video 1, Caplovitz et al., 2011).

We extended the application of the paradigm to address questions related to the binding problem by asking what happens if the objects are not identical—that is, if we provide additional identity information about the objects? Specifically, as shown in Figure 1B, we changed the features of the moving rectangles (for instance, instead of two black rectangles, we change one rectangle to red and the other to green). In this case, the color of the rectangles provides an additional, unambiguous source of information consistent with the two objects traversing from one side of the screen to the other, i.e., the streaming percept. We found that the visual system can rely on feature information in order to maintain existing object representations over space and time under some conditions, but not others. For example, while having distinct colors can lead to increases in perceived streaming, if the contrast of the two bars relative to the background was high and of the same polarity (i.e., red/green on a white background or a black background) then the bouncing percept would dominate.

Intriguingly, the conditions that produced bouncing also produced the percept of a dynamic unbinding and rebinding of the colors of the rectangles to which they belong. That is, as the rectangles appear to bounce off of each other, the colors simultaneously appear to switch from one rectangle to the other in an effect we call: “Feature Exchange.” Thus, the bar that had been red suddenly becomes green and visa versa. Here we show three new demonstrations of Feature Exchange that highlight the applicability of the effect to probe the binding problem across a wide range of perceptual domains. The demonstrations suggest that Feature Exchange can also be used to probe perceptual processes beyond the binding problem such as asymmetries between foveal and peripheral vision, the featural underpinnings of face perception, and the disambiguation of additional global motion trajectories. Specifically we show: (1) Colored rectangles that “bounce” (i.e., go from the end of the screen to the middle and then appear to return to the start) when viewed foveally, appear to “stream” when viewed peripherally. (2) Feature Exchange can occur with high-level visual objects such as faces (Face Exchange) that can differ across a range of facial categories (i.e., gender, race, etc.). (3) We introduce the “Feature/Face-Go-Round” paradigm in which two objects/faces, each of which moves only in one direction, are misperceived as moving in a circular pattern.

Taken together the new demonstrations show that in many conditions the brain defines feature-independent spatiotemporal correspondences (i.e., the motion) and then attaches the features to the objects—that is, define the motion and the objects will follow. As our past work has demonstrated, however, under certain conditions (i.e., when motion direction is ambiguous), both low-level and high-level features can contribute to maintaining pre-existing object representations. The Face-Exchange and Face-Go-Round paradigms can be used to systematically investigate the parameter space that determines how, if, and when features will contribute to the maintenance of object representations. In the spirit of this special issue on illusions and neuroscience, these paradigms represent valuable tools for probing a wide range of perceptual and cognitive questions offering neuroscientists a new approach for studying their underlying neural mechanisms.

## Demonstrations of feature exchange and their application

### Demonstration 1: feature exchange in the periphery

**Figure 2:**
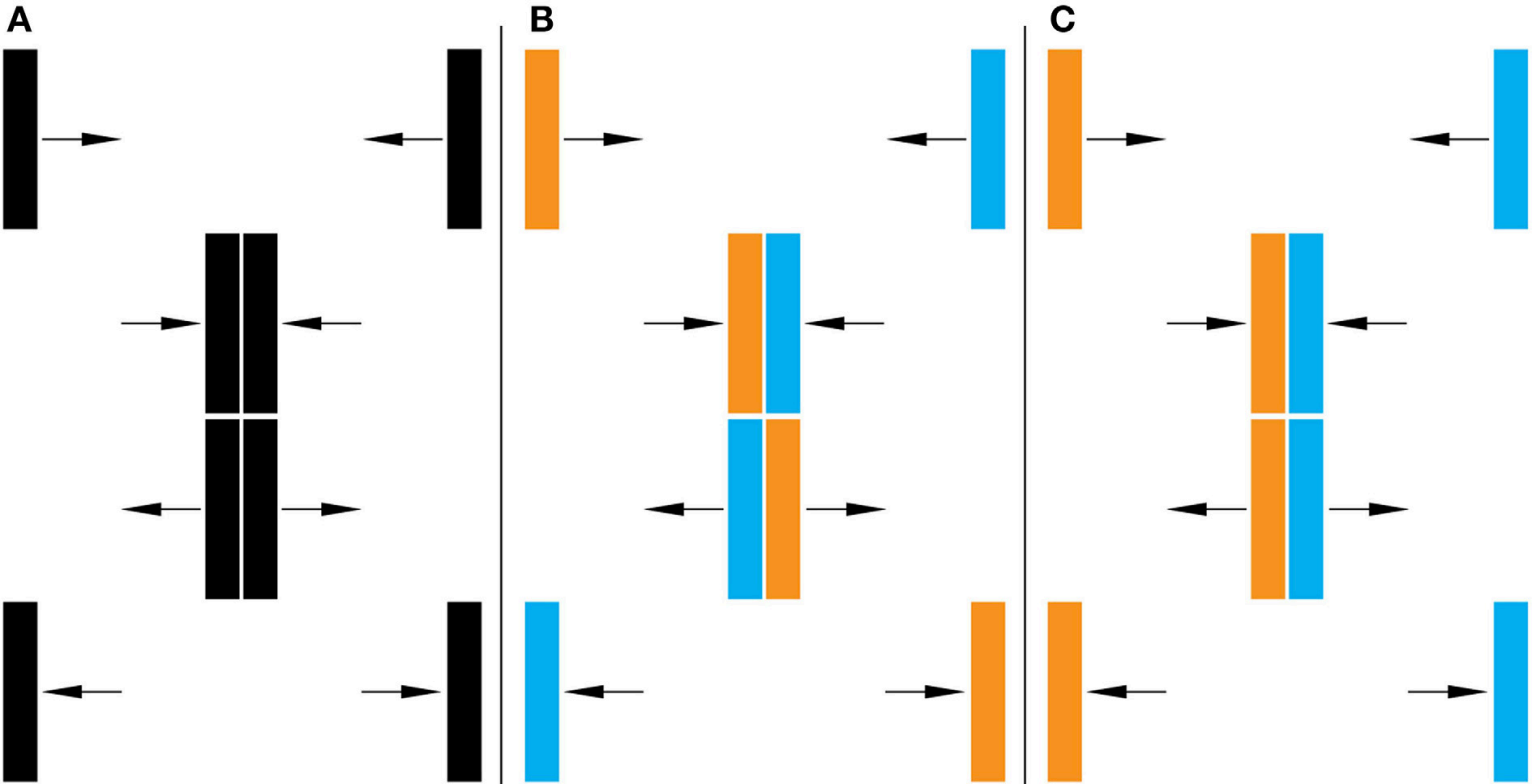
**Bounce/Stream Paradigm. (A)** Without distinct features. **(B)** Distinct features consistent with streaming, **(C)** features consistent with bouncing. When viewed foveally, all three tend to be perceived as bouncing, and streaming when viewed peripherally. Thus, Feature-Exchange occurs in **(B)** when viewed foveally and **(C)** when viewed peripherally. See Videos 1–4.

In our previous investigations, Feature Exchange was always directly linked to the bouncing percept. Namely, Feature Exchange would occur when the features were consistent with streaming, yet bouncing was perceived. Here we document the inverse can also be the case: Feature Exchange can also occur when the features are consistent with the bouncing percept and yet streaming is perceived. In this case, as each distinct object (i.e., each has a different color) is perceived to move from one side to the other, they exchange their features at the moment they stream past each other. Figure 2 illustrates a basic configuration with orange and blue bars. In Figure 2A, the bars are both the same color, so there is no physical difference between bouncing and passing. Although the stimulus is ambiguous, observers maintaining central fixation typically perceive the rectangles as bouncing (see Video 1 in Caplovitz et al., 2011). In Figure 2B, one bar is orange and the other blue, and the features of the bars are consistent with the streaming percept (see Video 1). In Figure 2C, the colors of the bars are consistent with the bouncing percept (see Video 2). As can be observed in the corresponding videos, when maintaining central fixation, both sets of colored rectangles tend to be perceived as bouncing, revealing the original Feature Exchange in Video 1. However, when the videos are viewed peripherally instead of maintaining central fixation, both tend to be perceived as streaming, revealing a novel manifestation of Feature Exchange in Video 2.

To document this effect, we showed the display to students (*N* = 26) at American University using a projector in a small classroom. The procedure was approved by the University's Institutional Review Board (IRB). Students were shown a series of videos and asked to view the display centrally and to view the display when looking to the side. Because our goal was to document a robust visual effect, we did not require specific measurements for the distance in the periphery; it would certainly be worth measuring such effects parametrically. The observers recorded whether they saw the display as bouncing or passing by circling their response on answer sheets provided prior to the start of the demonstration.

Table 1 lists the results as the percentage of observers who reported the bars as bouncing; for any cell, the value for perceived passing was always 100% minus the recorded perceived bouncing percentage. First let's consider the white background conditions. When the colors were consistent with bouncing, observers overwhelmingly (88%) reported that the bars appeared to bounce when viewed centrally, but appeared to pass when viewed peripherally (16% reported bouncing). Similar results were found when the colors passed each other: foveal viewing, 80% reported bounce (a result that replicates Caplovitz et al., 2011); peripheral viewing, 8% reported bounce.

**Table 1:** **Percent of observers reporting bars as bouncing**.

**Background**	**White background**		**Mid-luminance background**	
	**Central (%)**	**Periphery (%)**	**Central (%)**	**Periphery (%)**
Physically bounce	88	16	50	0
Physically pass	80	8	0	0

*Four conditions: white background, bars physically pass (Video 1), white background, bars physically bounce (Video 2), mid-luminance background, bars physically pass (Video 3), mid-luminance background, bars physically bounce (Video 4). Observers viewed the videos centrally and peripherally*.

We also presented observers videos with the bars on a mid-luminance background (see Videos 3, 4). The luminance level of the background was above the luminance of the blue bar and below the luminance of the orange bar, thus creating opposite contrast polarity between the bars and the background. We previously tested only the colors-passing condition, but showed that such a background completely eliminated any tendency for the bars to be perceived as bouncing (Caplovitz et al., 2011). Here, when the colors bounced, on central viewing half of the observers reported that the bars appeared to bounce, and when viewed peripherally all of the observers reported that the bars appeared to pass. As would be expected from Caplovitz et al. (2011), when the bars physically passed each other, all of the observers reported seeing the bars passing both when viewed centrally and peripherally.

The results can be interpreted in terms of the predominance of the motion signal over features. That is, on a white background, the motion of the bars appears to be bouncing when viewed centrally, and appears to be passing when viewed peripherally. Once this interpretation of the motion is instantiated, the features will follow the motion. On a mid-luminance background, the motion signal favors passing, therefore observers rarely see the bars as bouncing. Caplovitz et al. (2011) showed that the features can override the motion signal if the features are particularly salient. Still, 50% of the observers saw bouncing bars as passing (i.e., Feature Exchange), even when they were looking directly at them.

**Figure 3:**
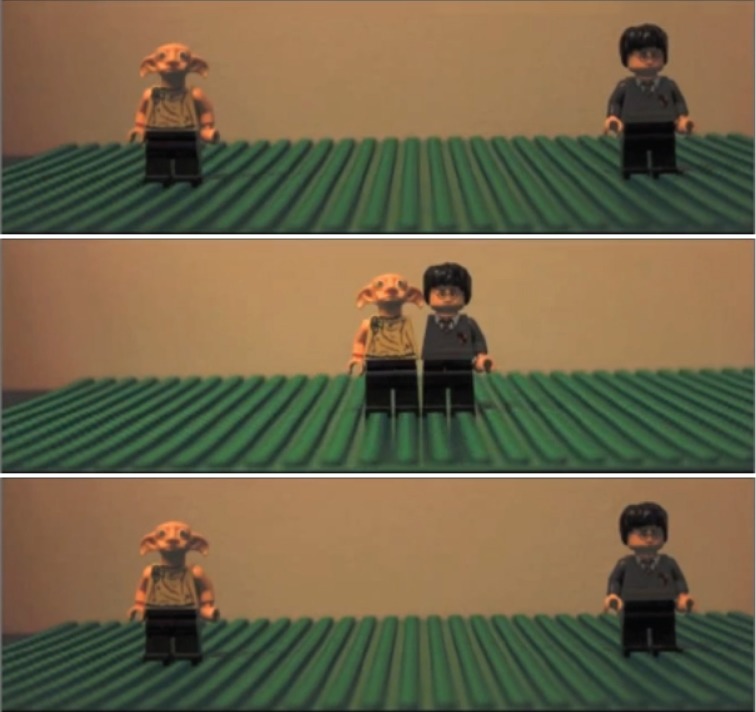
**Harry-Dobby illusion Demonstration 1 showed images that physically bounce appear to pass in the periphery**. Here we repeat the effect with stop action animation and toy characters. See Video 5.

Moreover, both bouncing and streaming manifestations of Feature Exchange can occur with more complex images of everyday objects. In one such example illustrated in Figure 3, two toy characters (A Harry Potter Lego figure and a Dobby Lego figure) move toward the center of the screen then return back to where they came from (i.e., bounce)—similar to Video 2. A stop action animation can be seen in Video 5. In this case, as with all the other examples, the objects bounce in the fovea and stream in the periphery. Depending on the distance from the screen and the size of the monitor, observers sometimes have to look far into the periphery in order to see the effect. Thus, the application of Feature Exchange to the study of asymmetries in foveal and peripheral processing is not limited to low-level features such as color or texture but can be extended to high-level complex image qualities. The next section emphasizes the utility of this point by demonstrating that Feature Exchange can occur with faces.

### Demonstration 2: face exchange

**Figure 4:**
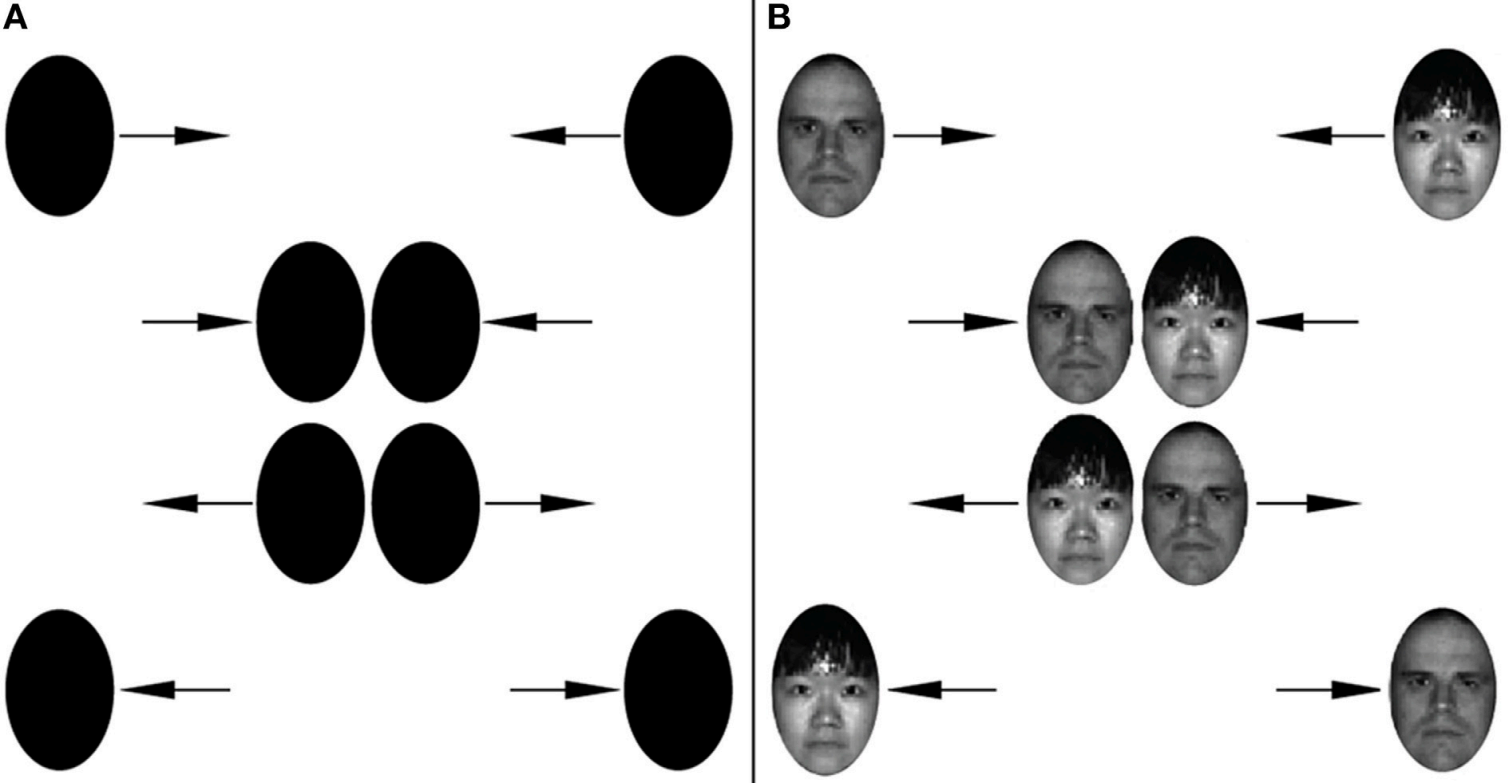
**Face-Exchange**. Identical ovals **(A)** are generally perceived to bounce. Under certain conditions, when the ovals are replaced with distinct faces whose identities are consistent with streaming **(B)**, observers will still perceive the bouncing percept. See Video 6 for **(A)** and Videos 7–9 to see faces from **(B)** on white, gray, or black backgrounds.

As illustrated in the Harry-Dobby illusion, the basic Feature Exchange paradigm can be applied with complex objects. Here we examine the effect with faces. In Figure 4A, two identical black ovals traverse from one side of the screen to the other. Because there are no featural differences between the two, the stimulus is equally consistent with both bouncing and streaming. As can be seen in Video 6 there is a tendency to perceive these ovals as bouncing. In Figure 4B, the ovals are replaced by two distinct faces. Despite the fact that the faces themselves are consistent with streaming (i.e., each face physically traverses from one side to the other), there is a tendency to continue to see the faces bounce off of each other, with the concomitant un-binding and rebinding of which face belongs to which moving object (Video 7). All face images in this paper are from the Extended Yale Face Database B (Georghiades et al., 2001).

To document the effect, we showed the videos to same participants as in Demonstration 1. Table 2 shows the results the faces against a white, mid-luminance, and black background. For all three backgrounds conditions, the face stimuli are perceived as bouncing by the majority of subjects when viewed foveally and passing when viewed peripherally (for mid-luminance and black backgrounds, see Videos 8, 9).

**Table 2:** **Percent of observers reporting faces as bouncing**.

**Background**	**Central (%)**	**Periphery (%)**
White	85	15
Mid-luminance	77	8
Black	88	31

*Three conditions: white background (Video 7); mid-luminance background (Video 8); black background (Video 9). Observers viewed the videos from each condition centrally and peripherally*.

Despite the intrinsic relevance of these results to the binding of complex features (i.e., facial characteristics), we note the general applicability of the demonstration as a tool to study the representation of faces in general. Over the past several decades, a great effort has been made to elucidate the mechanisms that underlie the representation of faces (Kanwisher, 2000; Haxby et al., 2002; Kanwisher and Yovel, 2006; Gobbini and Haxby, 2007). Based on the study of adaptation and so-called face-aftereffects (Webster and MacLeod, 2011), there is a growing consensus that faces are represented according to a multidimensional face-space. A given face may be represented by its unique location along myriad dimensions that may be configural (Webster and MacLin, 1999) or more conceptual such as age, gender, emotion, race, etc. (Webster et al., 2004). Despite the growing consensus about the existence of a face space, a challenge remains in identifying what the actual dimensions that define the space are and how these dimensions interact with each other (Said and Todorov, 2011). We suggest that Feature Exchange represents a new and potentially valuable tool for investigating these questions. Just as we were able to use the rate of Feature Exchange to probe interactions between low-level features such as contrast, color and texture, the same approach could be applied to the study of face-space dimensions (i.e., two faces that differ on one dimension lead to similar rates of Feature Exchange as two faces that differ on another).

### Demonstration 3: feature exchange with the Go-Round paradigm

The previous examples with the bounce/pass paradigm suggests that even for complex objects (faces, Lego characters), situations can be created in which the way the visual system resolves conflicts between the trajectories of objects and their defining features leads to Feature Exchange. Here we create a different form of the illusion based on the same principle between the juxtaposition between object constancy and motion flow, only this version does not require that the objects meet at a point of intersection.

**Figure 5:**
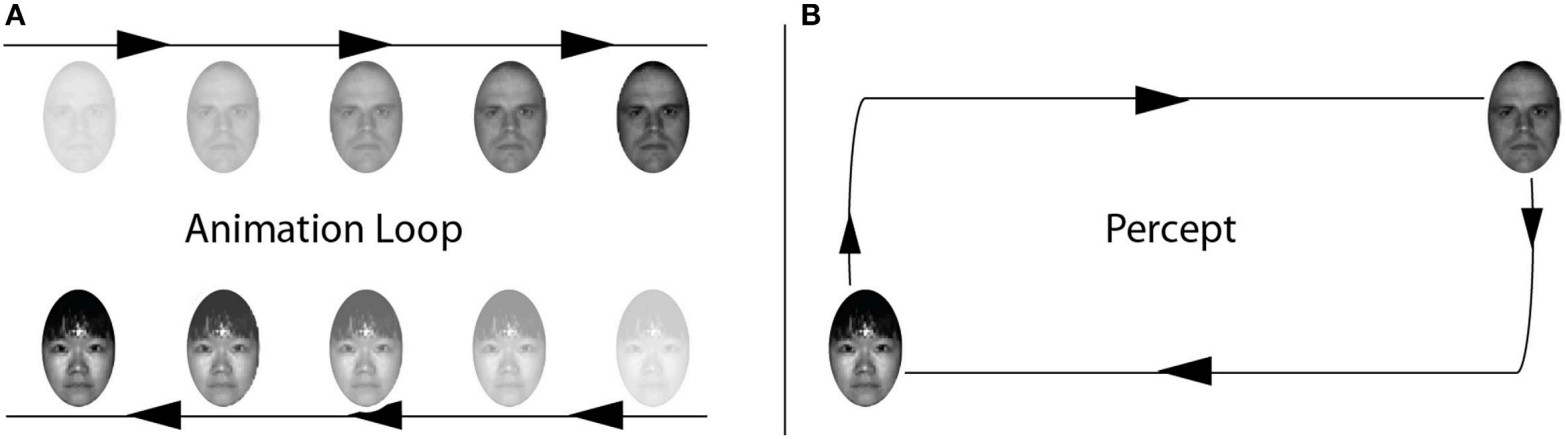
**Face-Go-Round. (A)** Two faces repeatedly traverse from one side of the screen to the other. Rather than perceive each face repeatedly traversing the same linear trajectory, under certain conditions observers tend to see each face circling around each other over the same trajectory **(B)**. See Video 10.

As illustrated on the left of Figure 5, the stimulus consists of two objects—in this case the same faces as that used in Demonstration 2. Face 1 starts near the top left portion of the screen and slides to the right; when the face reaches the right portion of the screen, it disappears and then reappears at the initial starting point and continues its move to the right. Face 2 does the opposite motion at the bottom of the screen—that is, the face starts bottom right and slides to the left; when it reaches left portion of the screen it disappears and then reappears at its initial position and continues to slide.

The spatiotemporal correspondence provided by the disappearance and reappearance of the faces is ambiguous. For example, when Face 1 disappears at the top right of the screen, it is followed immediately by (A) the appearance of Face 1 at the top left, and (B) the appearance of Face 2 at the bottom right. The visual system could, in principle, form an object correspondence between the disappearance of Face 1 and either of these two locations. The advantage of interpretation A is that it provides object constancy; the advantage of B is that it provides the closest motion signal–and it has been shown that in ambiguous motion conditions the closest signal tends to predominate. So, just like in the standard feature exchange paradigm we are confronted with two conflicting hypotheses. As can be observed in Video 10, the go-round percept is readily observed, along with the corresponding exchange of facial identities (Figure 5-Right).

Depending on the question being investigated, this paradigm for examining Feature Exchange may offer distinct empirical advantages over the one described in Demonstrations 1 and 2. Specifically, because the exchange of features does not depend on a point of intersection, the Go-Round paradigm avoids issues related occlusion, depth ordering and a whole host of issues that manifest at the point of intersection in the bounce/stream paradigm (Michotte, 1946/1963). Moreover, because the feature exchange occurs symmetrically in the display, the paradigm is more amenable to parametric manipulations of spatial factors, particularly those related to eccentricity and the visual field.

## Discussion

We write this article for a special issue on visual illusions and their application to the study of human neuroscience. The term “illusion” is a notoriously difficult category; the purpose of “illusions” in visual sciences has been debated for centuries (see Boring, 1942). We are not going to venture into these arguments. Instead we hope our paper will highlight one particular approach for applying illusions to the study of visual perception and human neuroscience that for the most part can serve as a guiding principle for the generation of new visual phenomena. We believe that illusions can be particularly informative, and most easily created, when they address questions about how the visual system works; that is, we aim to produce research-generated phenomena. Even though the phenomenological aspects of such illusions may be of interest to naive observers, they are fundamentally of import because they address particular questions about visual processing and can thus either directly elucidate underlying neural mechanisms of visual perception or be used as empirical tools for their study. In this respect, therefore, the phenomenology of such illusions can be considered as superthreshold experiment or explorations of a particular idea.

The demonstrations presented here were born out of our interest in questions related to the binding-problem—what allows the visual system to create and maintain representations of objects, even though all indications show the human brain contains multiple separable streams of processing? We approached this question by examining how perception resolves conflicts between information about the position and motion of objects and feature information about their surfaces. Although the demonstrations of Feature Exchange grew organically out of these inquiries, once observed, their phenomenology revealed new potential applications for how other challenging questions may be approached. These applications include but are not limited to investigations into the behavior and neural mechanisms underlying asymmetries in foveal/peripheral processing of objects and features, similarities, differences and interactions between the processing of low- and high-level feature information (Webster and MacLin, 1999; Said and Todorov, 2011), theories of object formation and tracking (Pylyshyn and Storm, 1988; Kahneman et al., 1992; Flombaum et al., 2004, 2009; Feldman and Tremoulet, 2006; Mitroff and Alvarez, 2007) and interactions between different motion processing systems (i.e., those that operate on luminance contrast or other spatiotemporal changes in the visual scene) (Cavanagh, 1992; Lu and Sperling, 1995, 2001).

### Conflict of interest statement

The authors declare that the research was conducted in the absence of any commercial or financial relationships that could be construed as a potential conflict of interest.
